# Is there an association between spatial accessibility of outpatient care and utilization? Analysis of gynecological and general care

**DOI:** 10.1186/s12913-018-3143-5

**Published:** 2018-05-03

**Authors:** Ulrike Stentzel, Jeanette Bahr, Daniel Fredrich, Jens Piegsa, Wolfgang Hoffmann, Neeltje van den Berg

**Affiliations:** 1 Institute for Community Medicine, University Medicine Greifswald, Ellernholzstraße 1-2, 17487 Greifswald, Germany; 2 Clinic and Outpatient Clinic for Internal Medicine C, University Medicine Greifswald, Sauerbruchstraße, Diagnostic Center, 17475 Greifswald, Germany

**Keywords:** Accessibility, Utilization, GP, Gynecologist, GIS

## Abstract

**Background:**

In rural regions with a low population density, distances to health care providers as well as insufficient public transport may be barriers for the accessibility of health care. In this analysis it was examined whether the accessibility of gynecologists and GPs, measured as travel time both by car and public transport has an influence on the utilization of health care in the rural region of Western Pomerania in Northern Germany.

**Methods:**

Utilization data was obtained from the population based Study of Health in Pomerania (SHIP). Utilization was operationalized by the parameter “at least one physician visit during the last 12 months”. To determine travel times by car and by public transport, network analyses were conducted in a Geographic Information System (GIS). Multivariate logistic regression models were calculated to identify determinants for the utilization of gynecologists and GPs.

**Results:**

There is no significant association between the accessibility by car or public transport and the utilization of gynecologists and GPs. Significant predictors for the utilization of gynecologists in the regression model including public transport are age (OR 0.960, 95% CI 0.950–0.971, *p* < 0.0001), social class (OR 1.137, 95% CI 1.084–1.193, p < 0.0001) and having persons ≥18 years in the household (OR 2.315, 95% CI 1.116–4.800, *p* = 0.0241).

**Conclusions:**

In the examined region less utilization of gynecologists is not explainable with long travel times by car or public transport.

## Background

In sparsely populated rural regions the spatial distribution of healthcare providers cannot be as dense as in urban regions. A possible consequence could be that the spatial accessibility of medical facilities [1] affects the utilization of health services. Spatial accessibility refers to the ease to reach e.g. medical services and facilities [2, 3]. The definition of ease includes both the geographic distance that must be overcome and the needed time to do so. Large distances to healthcare [4–11] as well as insufficient public transport [12–14] may be barriers for access to and utilization of healthcare facilities. Different approaches have been used to study the accessibility. Some studies draw on physician density [15, 16], some on distances assessed in self-reports [4, 13], some took distances from web resources which provide these information [6]. Simplified methods include calculation of straight-line distances (Euclidian) in Geographic Information Systems (GIS) [9, 12, 17]. However, several studies used exacter geographic methods [5, 7, 8, 11, 15, 18–20]. Most GIS allow performing network analyses that calculate distance in length measurement units or in time units based on routable road network data. Commonly the above mentioned analyses confined to accessibility by motorized individual transport (cars or other motorized vehicles). The motorized individual transport plays the essential part in traffic in rural regions, but not everyone in Germany has access to a private car [21]. The population in Germany’s rural regions is often older and the regional deprivation is higher [22, 23]. Hence, vulnerable parts of the population might be dependent on public transport. However, in German rural regions public transport usually is orientated to school traffic [24]. Therefore, accessibility by public transport should be considered [25]. Only a few studies considered accessibility by public transport. This is mostly due to the lack of appropriate public transport data for the use in GIS. Studies that included public transport used either self-reported data or defined the quality of accessibility by the frequency (high versus low) of public transport services or whether or not a section of the rural road network was bus serviced at least every hour during daytime [8, 14, 20].

The objective of this analysis was to examine whether accessibility by car and by public transport is associated with the utilization of outpatient general practitioners (GP) and gynecologists as an example for specialized physicians.

In contrast to many other countries, the people in Germany have the free choice of doctor, which is incorporated into law. People with a disease or a health problem normally have to consult an outpatient general practitioner (GP) at first. For particular medical problems, the patients will be referred to an outpatient medical specialist. In some cases, e.g. for routine examinations at gynecologists, patients can also go directly to medical specialists without referral [26].

The study region was the region Western Pomerania in the northeast of Germany. This region has 322,863 inhabitants, a mean population density of 77 inhabitants per km^2^ (12/2010 [27]) and is classified as a rural area of lower population density [28]. The research question was that the utilization of GPs and gynecologists is lower with longer travel time to the nearest GP/gynecologist.

## Methods

### Data

For reasons of comparability, both analyses were focused only on women. Utilization, age, socioeconomic data and persons in the household ≥18 years were retrieved from the 5-year follow-up (SHIP-1) of the population-based epidemiologic cohort study “Study of Health in Pomerania” (SHIP) which was performed in 2002–2006 [29]. SHIP-1 contains in total 3300 participants. Thereof, 1172 female participants were included in the analysis. The participants were of age 25–88. Therefore, only the utilization of adults is considered. In SHIP-1 the utilization of GPs and gynecologists during the twelve months prior to the data assessment was obtained in a standardized face-to-face interview. Participants self-reported at least one visit in the 12 months prior to the assessment. The Winkler social class index was used to represent social status [30, 31]. The social class index is a multi-dimensional index that considers income, education, and professional status [32–34].

Persons in the household ≥18 years were included, because they are old enough to have a driver’s license and might be able to give a ride. A comprehensive description of the SHIP study is published by John et al. [29].

### Calculation of distances and travel times

The spatial accessibility of the physician practices was operationalized by calculating travel times between the residential addresses of each participant and the nearest practices by car and by public transport. For both analyzes was an underlying assumption that the participants use the respective transportation mode and that they visit the closest practice.

Geographical coordinates of the residential addresses of the female participants of SHIP-1 and the locations of the practices of 248 GPs and 38 gynecologists in the study region were calculated in a geographic information system (GIS) (ESRI®ArcGIS™ 10.0 Esri Inc., Redlands/California (USA)). Additionally, 93 GPs and 9 gynecologists in a 12 km buffer zone around the study region were included. The addresses of the practices were retrieved from the physician directory of the Association of Statutory Health Insurance Physicians of the federal states of Mecklenburg-Western Pomerania and Brandenburg.

### Travel times by car

The distances between the residential addresses of the participants and the physician practices and the travel times by car were calculated using routable digital street data (Dplus, Logiball, Herne, Germany). The calculation of distances and travel times was conducted with ArcGIS, using the software extension Network Analyst which allows network-based spatial analysis. Not considered were delays due to traffic jams, construction sites etc.

### Travel times by public transport

The accessibility of the practices by public transport was calculated on the basis of timetables of busses and trains. Geographic coordinates of bus and train stops were partly delivered by public transportation services and partly assessed in the field by project staff with GPS devices.

The calculation of the accessibility by public transport included some assumptions:

- The appointment with the physician was set at Tuesday, 11 am during school times, because public transport is closely aligned with the transport of school children.
- The pedestrian speed was set at 4 km/h for SHIP-1 participants under 51 years, 3 km/h for 51–69 year old participants and 2 km/h for participants of 70 years and older.
- Foot walks between the patients homes and the practices and between the homes of the patients and the bus and train stops were limited to a maximum length of 1000 m.
- Foot walks between the bus and train stops and the practices were limited to a maximum length of 500 m.
- Foot walks needed to change between busses and/or trains were limited to a maximum length of 250 m.
- The journey back home after the physician appointment was set to start at 12 pm and had to be finished the same day before midnight.

The duration of the appointment with the physician was not included in the travel time. The calculated travel time was the total travel time of the roundtrip.

The calculation of the travel times by public transport was conducted on the basis of the Dijkstra algorithm [35, 36] using a self-developed network analysis software [25]. The shortest travel time (in minutes) was determined along the nodes of the network and their connecting edges. The footpaths were calculated with the network Analyst in ArcGIS.

### Statistical analysis

Multivariate logistic regression analyses were conducted to identify determinants for the utilization of gynecologists and GPs. Outcome of the regression analyses was the utilization of the gynecologist and/or GP in the 12 months prior to the assessment (operationalized as at least one visit in the 12 months prior to the assessment). Age was included as a metric variable. The travel times were included as a metric variable in minutes by car and as a categorical variable (categories: ≤ 60 min (min), > 60 – ≤ 120 min, > 120 - ≤ 180 min, > 180 min, no connection at all) by public transport. The social class index is calculated additively as a point sum score [37] and was also included as a metric varaible. The higher the score the higher the social status. SAS 9.3 © 2002–2010 (SAS Institute Inc., Cary, NC, USA) was used to perform all statistical analyses.

## Results

**Fig. 1:**
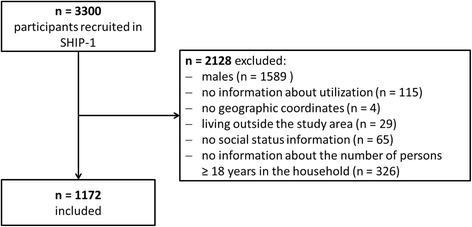
Number of patients included in the analysis

SHIP-1 has 3300 participants in total. The flowchart in Fig. 1 shows the number of included and excluded participants in this analysis and the reasons for exclusion.

### Utilization

**Fig. 2:**
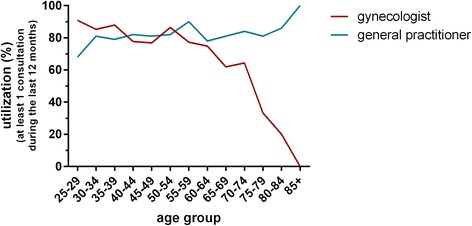
Utilization of gynecologists and GPs by women in SHIP-1 in the 12 months prior to the assessment (at least one visit)

Figure 2 shows the utilization of gynecologists and GPs by women in SHIP-1 by age group during the 12 months prior to the assessment. The utilization of GPs increases with increasing age whereas the utilization of gynecological medical care is high in young age groups and decreases with increasing age.

### Spatial accessibility

#### General medical care

**Fig. 3:**
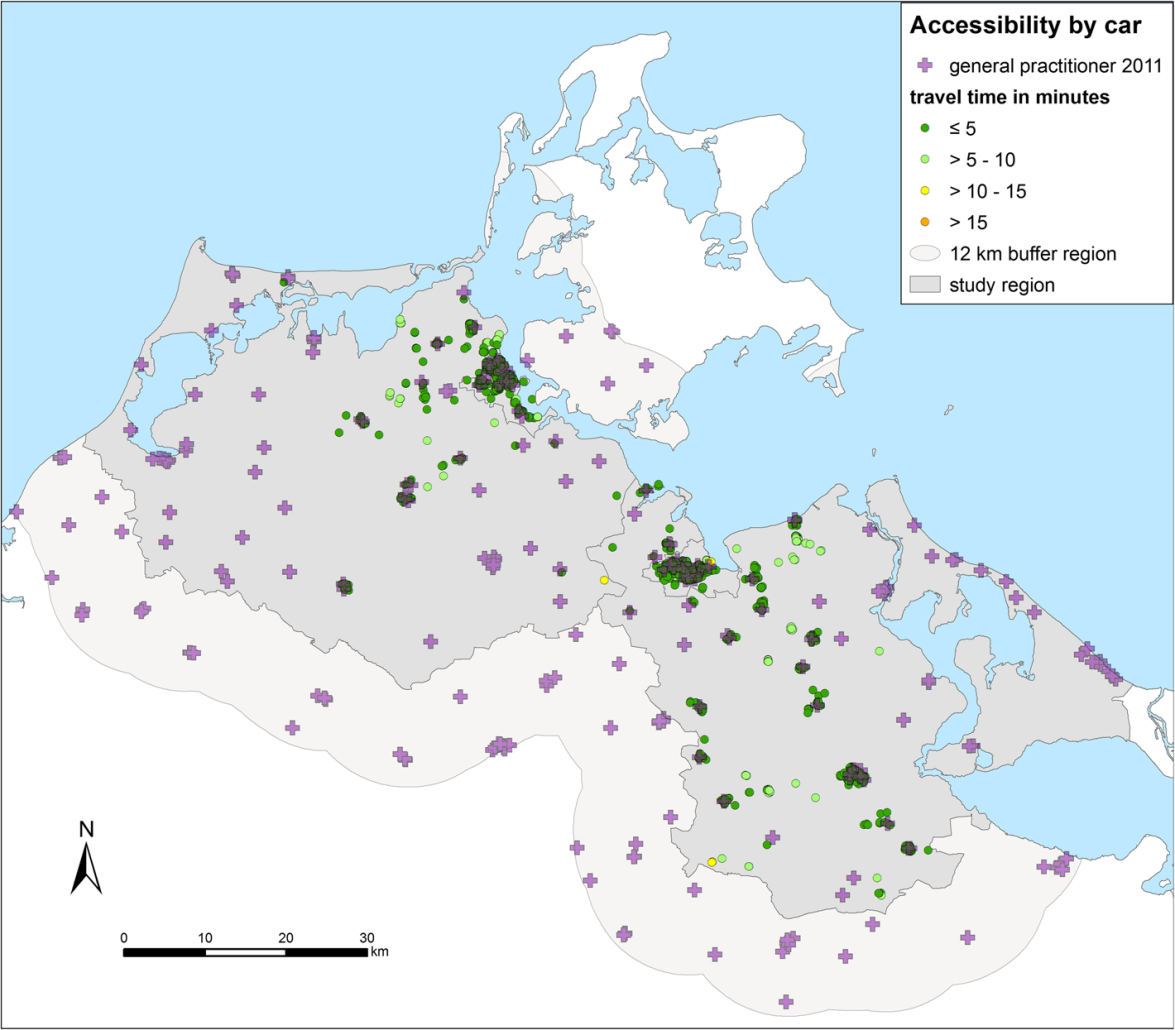
Accessibility of GPs by car. Travel times from the homes of the patients to the nearest practice in minutes (one way) in the region Western Pomerania in the northeast of Germany (source: author’s own figure/map)

Figure 3 shows the results of the geographical analysis of the accessibility of GPs by car. Table 1 shows the number of patients in the different travel time categories. More than 90% of the participants have a travel time of 5 min or less.

**Table 1:** Accessibility of GPs by car: number and proportion of participants, mean and standard deviation (SD) of travel time to the nearest practice

Travel time in 5-min-categories	Participants			
	number	%	mean	SD
≤5 min	1079	92.1	1.3	1.0
> 5–10 min	87	7.4	7.1	1.7
> 10–15 min	6	0.5	11.3	0.9

**Fig. 4:**
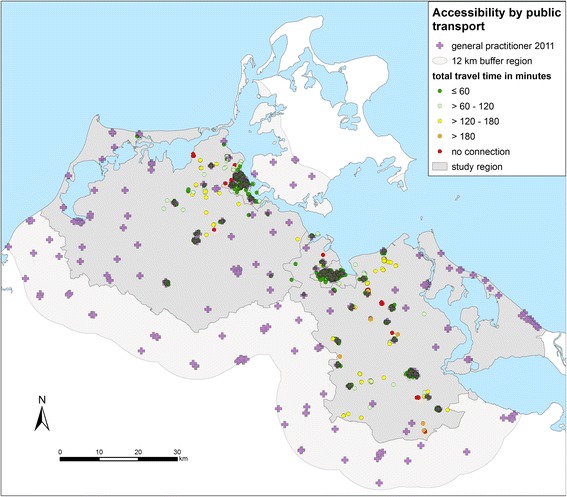
Accessibility of GPs by public transport from the homes of the patients to the nearest practice in minutes (round trip) in the region Western Pomerania in the northeast of Germany (source: author’s own figure/map)

The accessibility with regard to public transport differs from the accessibility by car (Fig. 4). 83% of the participants (*n* = 968) of the study participants have access to a GP within one hour by public transport (Table 2) whereas 2.5% of the participants (*n* = 29) have no access with public transport. This means that they can’t travel to the nearest GP and travel back within one day. The distance to the practices does not play a major role. Accessibility rather depends on the connection to the public transport network.

**Table 2:** Accessibility of GPs by public transport: number and percentage of participants, mean and Standard deviation (SD) of travel time to the nearest practice

Travel time in 60-min-kategories	Participants		Travel time (minutes)	
	number	Percent (%)	mean	SD
≤60	968	82.59	17.2	11.8
> 60–120	77	6.57	96.3	17.3
> 120–180	87	7.42	146.5	16.8
> 180	11	0.94	198.7	10.2
No connection	29	2.47	–	–

#### Gynecological practices

**Fig. 5:**
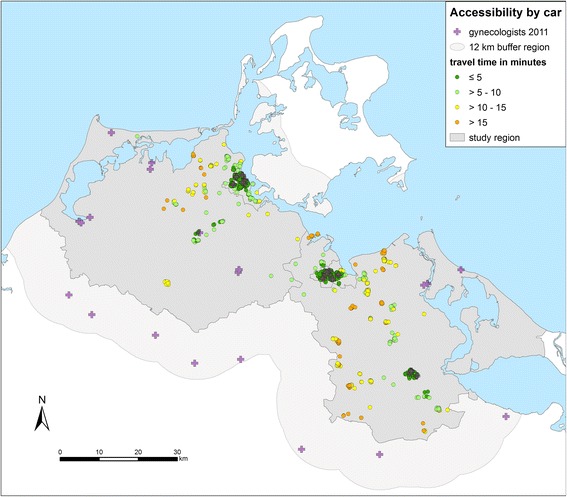
Accessibility of gynecologists by car. Travel times from the homes of the patients to the nearest practice in minutes (one way) in the region Western Pomerania in the northeast of Germany (source: author’s own figure/map)

Compared to GPs the number of the gynecologists as an example for specialized physicians is much lower and their spatial distribution in the region is less dense. This is reflected in longer travel times by car (Fig. 5 and Table 3). Although the gynecological practices are concentrated in the larger towns, just 63.6% of the participants have a travel time of 5 min or less.

**Table 3:** Accessibility of gynecologists by car: number and percentage of participants, mean and standard deviation (SD) of travel times to the nearest practice

Travel time in 5-min-categories	Participants			
	number	%	mean	SD
≤ 5 min	745	63.57	1.8	1.0
> 5–10 min	165	14.08	7.9	1.5
> 10–15 min	169	14.42	12.5	1.6
> 15 min	93	7.94	17.3	2.7

**Fig. 6:**
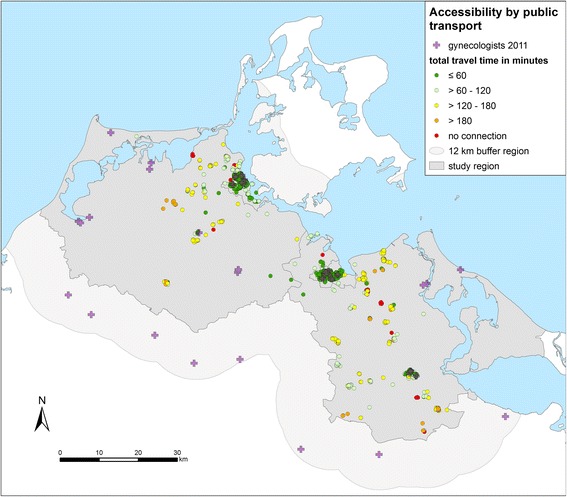
Accessibility of gynecologists by public transport from the homes of the patients to the nearest practice in minutes (round trip) in the region Western Pomerania in the northeast of Germany (source: author’s own figure/map)

The accessibility by public transport is also poorer for gynecologists than for GPs (Fig. 6 and Table 4). Just 62.5% of the participants have access within one hour. The participants without access by public transport to the nearest gynecologists are the same as in the analysis of the accessibility of the GPs.

**Table 4:** Accessibility of gynecologists by public transport: number and percentage of participants, mean and standard deviation (SD) of travel time to the nearest practice

Travel time in 60-min-categories	Participants			
	number	%	mean	SD
≤60 min	733	62.5	25.4	12.6
> 60–120 min	184	15.7	93.2	15.3
> 120–180 min	182	15.5	146.6	16.2
> 180 min	44	3.8	204.1	18.6
No connection	29	2.5	–	–

### Logistic regression models

#### General practitioners

Regarding GPs, only the social class index was found to be a significant determinant for utilization in both models. If the social class index increases by 1, the probability of utilization decreases by a factor of 0.947 (by car, Table 5) or 0.948 (by public transport, Table 6). The odds for utilization of GPs increase with increasing age and with increasing travel time, but the coefficients are not statistically significant. However, regarding travel time a trend is clearly present. The odds for utilization decrease with persons in the household ≥18 years, but again the association does not reach the level of statistical sign (Table 5 and Table 6).

**Table 5:** Multivariate logistic regression analysis of the influence of travel time by car on the utilization of GPs, *n* = 1172 participants (SAS proc. logistic)

Predictor	*β*	*SE β*	*p (α = 0.05)*	*e*^*β*^ (odds ratio)	CI	
					2.5%	97.5%
Travel time by car (min.)	0.0828	0.0446	.063	1.09	0.995	1.186
Age	0.00756	0.00585	.197	1.01	0.996	1.019
Social class index^a^	−0.0543	0.0264	.040	0.95	0.899	0.997
Persons ≥18 years in the household (yes/no)	−0.3214	0.4576	.482	0.73	0.296	1.778

*Abbreviations: β,* regression coefficient; *SE*, standard error; *CI* confidence interval; ^a^ Winkler social class index (Winkler, 1998, Winkler and Stolzenberg, 1999)

**Table 6:** Multivariate logistic regression analysis of the influence of travel time with public transport on the utilization of GPs. *n* = 1172 participants (SAS proc. logistic)

Predictor	*β*	*SE β*	*p (α = 0.05)*	*e*^*β*^ (odds ratio)	CI	
					2.5%	97.5%
Travel time with public transport*						
no connection	−0.1484	0.4566	.745	1.14	0.429	3.050
*t* > 180 min	0.4343	0.8525	.610	2.05	0.259	16.227
120 min < t ≤ 180 min	0.0642	0.3473	.853	1.41	0.751	2.667
60 min < *t* ≤ 120 min	−0.0669	0.3490	.848	1.24	0.655	2.353
Age	0.00720	0.00584	.218	1.017	0.996	1.019
Social class index^a^	−0.056	0.0264	.043	0.95	0.900	0.998
Persons ≥18 years in the household (yes/no)	−0.3161	0.4573	.489	0.73	0.298	1.786

*reference travel time by public transport *t* ≤ 60 min Abbreviations*: β,* regression coefficient; SE, standard error; CI confidence interval, t, travel time; ^a^ Winkler social class index (Winkler, 1998, Winkler and Stolzenberg, 1999)

#### Gynecological care

Regarding the travel time by car, the probability of utilization of a gynecologist decreases when the travel time by car is higher, but this difference is not statistically significant (Table 7). With respect to public transport, the probability of utilization decreases when the travel time by public transport is above 60 min compared to travel times less or equal than 60 min (Table 8), but the coefficient is not statistically significantly different from the Null and the confidence interval (CI) is large.

**Table 7:** Multivariate logistic regression analysis of the influence of travel time with car on the utilization of gynecologists, *n* = 1172 (SAS proc. logistic)

Predictor	*β*	*SE β*	*p (α = 0.05)*	*e*^*β*^ (odds ratio)	CI	
					2.5%	97.5%
Travel time by car (min.)	−0.0160	0.0131	.223	0.98	0.959	1.010
Age	−0.0412	0.00573	.000	0.96	0.949	0.971
Social class index^a^	0.1304	0.0244	.000	1.14	1.086	1.195
Persons ≥18 years in the household (yes/no)	0.8443	0.3724	.023	2.32	1.121	4.826

*Abbreviations: β,* regression coefficient; *SE*, standard error; *CI* confidence interval; ^a^ Winkler social class index (Winkler, 1998, Winkler and Stolzenberg, 1999)

**Table 8:** Multivariate logistic regression analysis of the influence of travel time with public transport on the utilization of gynecologists. n = 1172 participants (SAS proc. logistic)

Predictor	*β*	*SE β*	*p (α = 0.05)*	*e*^*β*^ (odds ratio)	CI	
					2.5%	97.5%
Travel time with public transport^a^						
no connection	−0.1518	0.3557	.670	0.69	0.292	1.645
t > 180 min	−0.2859	0.2821	.311	0.61	0.310	1.185
120 min < t ≤ 180 min	0.0817	0.1844	.658	0.88	0.587	1.303
60 min < t ≤ 120 min	0.1404	0.1867	.452	0.93	0.619	1.389
Age	−0.0405	0.00574	.000	0.96	0.950	0.971
Social class index^b^	0.1286	0.0245	.000	1.14	1.084	1.193
Persons ≥18 years in the household (yes/no)	0.8394	0.3721	.024	2.32	1.116	4.800

^a^reference travel time by public transport t ≤ 60 min Abbreviations*: β,* regression coefficient; SE, standard error; CI confidence interval;, t, travel time; ^b^ Winkler social class index (Winkler, 1998, Winkler and Stolzenberg, 1999)

The odds for utilization declines in both models when age is increasing (travel time by car and by public transport). In contrast, the odds for utilization increase with increasing social class index. The odds for utilizing a gynecologist are two times higher if the participants live together with persons ≥18 years in their household (Table 7 and Table 8).

## Discussion

The hypothesis of this analysis was that the utilization of GP and medical specialist care (here: gynecologists) would be influenced by the travel times to the practices. A trend can be seen, but the results show, that neither travel time by car nor by public transport is significantly associated with whether or not a gynecologist or GP was consulted in the previous 12 months. Some international studies found associations between spatial accessibility and utilization or issues like regular check-up visits. Jones et al. [20] and Celaya et al. [17] found associations between tumor stage at diagnosis of breast cancer and increasing travel time to GP and other medical services. Jones et al. could prove for breast and colorectal cancers that the availability of public transport was associated with a reduced risk of late stage diagnosis. The authors assume that because of longer travel times patients were less likely to make doctor’s appointments compared to people living closer to the providers [20]. Arcury et al. [8] found that greater distance resulted in fewer regular check-up visits while distance was not significant in determining the number of chronic care and acute care visits. More important than distance for adherence to regular check-up visits was holding a driver’s license. Users of chronic care visits showed a significantly higher number of uses of shared rides and public transport. Even though these authors determined that distance was not a major barrier to chronic care they conclude that a lack of access to transportation may lead to less utilization of medical care [8]. Rocha et al. examined the role of public transport in accessibility to emergency dental care in Melbourne. They revealed that a similar number of patients came from areas with and without access to public transport. At least for emergency dental care access to public transport had no effects on utilization [14]. A study among American Indian and Alaska Natives in the United States showed that the geographic location of patients, their potential access to cancer screening services and utilization of cancer screening are all interrelated [19]. The authors showed that the proportion of cancer screened females dropped significantly with a higher degree of rurality and greater distance to the nearest provider.

Two other studies came to the result that distance to the closest mammography facility is indeed a significant risk factor for predicting advanced stage diagnosis in breast cancer [38, 39]. A Norwegian study could show an association between increasing distance to the primary care clinic and lower utilization of out-of-hours services except for telephone consultations [11]. Another study found that a reduction of the distance or the travel time to a hospital leads to an increase of the utilization of hospitals by COPD patients [40]. The results of all these studies indicate that travel time has an influence on the utilization of health care providers. In contrast, Tarlov et al. [41] found no significant association between distance to mammography facilities and the tumor stage at diagnosis.

The actual use of public transport in our study region is rather low. A survey of the National Association of Statutory Health Insurance Physicians in 2010 found that only 8% of the respondents used public transport [42]. A recent survey in an adjacent and very similar rural region in Germany determined that 67.7% of the participants used the car to get to a doctor’s appointment and 26% used the bus [43].

As a consequence of the low population density, the public transport network is often poor in rural regions, which in turn tends to reduce the number of users [44]. Where public transport is inadequate, a large proportion of peripherally resident people apparently are used to drive by car or find other solutions like rideshares to run their errands. This seems to include also their visits to GPs and specialist physicians. Having persons ≥18 years in the household was a significant determinant for the utilization of gynecologists. In our analysis this suggests that for older women living more remote, the utilization of gynecologic facilities may depend on the opportunity to get rides to the gynecologist. Travel time is obviously not decisive to explain the lower utilization of gynecologists in the study region.

Living with other adults in the household was not a significant predictor for the utilization of GPs. It may generally be easier to reach the GP because of the larger geographic density of GP-practices.

The results show that the utilization of gynecologists significantly increased with higher social class index. Higher education might foster higher health competence and awareness for health checks [45]. Data from the cross-sectional German Health Servey (Gesundheit in Deutschland aktuell, GEDA) showed an association between low check-up participation and low social status [46]. That study detected that education isn’t significantly associated to check-up participation, but occupational status and income are. An evaluation of the German Health Survey in the German federal state North Rhine-Westphalia found that women from the upper class have higher participation rates for cancer screening tests than women from the lower class [47]. On the contrary an evaluation of class-specific utilization of medical services and prevention in Bavaria in the south of Germany did not show differences between socioeconomic classes regarding mammography and other cancer screening tests [48]. Tian et al. tried to identify risk factors for disparities in breast cancer mortality among African-American and Hispanic Women [49]. They concluded that access to mammography facilities does not determine a greater utilization. A lower SES, however, was associated with lower utilization of mammography facilities. This corresponds to our findings regarding the utilization of outpatient gynecological care. Also the fact that the utilization of GPs decreases with higher social class index is corresponding with other studies [50–54].

### Limitations and strengths

The determination of accessibility is based on a range of assumptions. For example it was assumed that patients get treatment from the geographically nearest physician’s practice. However, accessibility is not always the determining criterion for the choice of one’s doctor. Also qualifications, reputation and recommendation [55] of an individual physician, availability of appointments as well as other activities of daily life (e.g. work locations) can influence the choice of a doctor [56].

The accessibility by car can be calculated under relatively realistic conditions whereas regarding the public transport accessibility requires several assumptions. These include the length of the foot paths and walking speed. These definitions, in part, determine which patients are assigned to the category “no connection”. The doctor’s appointment (Tuesday, 11 am during school time) was chosen for all calculations because this was the time with best public transport connections. Both at other times and during the holidays connectivity was found to be lower.

Unfortunately, we did not know whether the participants use the car or public transport. A cross-sectional survey assessed the mobility behavior of people aged 60 years and older in the same region as our study region. 33.7% of the participants reported, that they usually walk, use a bike, the bus, or other modes of transportation for the study region [57].

Major sickness as a covariate is not considered in the model. Chronic diseases would be a good parameter for the model. The SHIP data does not provide an appropriate variable. Besides all women are recommended to regularly visit a gynecologist for preventative check-ups. Therefore, we accepted this limitation.

The model fit was evaluated with goodness of fit statistic (Hosmer-Lemeshow test). It was computed for all four models. Both models regarding GPs are acceptable models for the data with *p*-values of 0.8273 (car) and 0.9188 (public transport). The p-value of the gynecologists-model are slightly below and above alpha 0.05 with 0.0333 (car) and 0.0547 (public transport). The goodness of fit is marginally to low here.

Strengths of the study are (a) the data from the population based prospective SHIP-cohort and (b) the consideration of real road-networks (including specific speed categories for motorized vehicles as well as for pedestrians), real bus and train schedules and changes of means of transport. Thus, the model uses valid data and depicts the reality in the study region as close as possible.

## Conclusions

This study showed that accessibility is not significantly associated with the utilization of GPs and gynecologists in a rural region of Western Pomerania. Significant predictors for the utilization of gynecologists are social class index and persons ≥18 years living in the household of the participants.

## Abbreviations

GIS: Geographic information system
GP: General practitioner
SHIP: Study of Health in Pomerania
